# Effectiveness between Dry Needling and Ischemic Compression in the Triceps Surae Latent Myofascial Trigger Points of Triathletes on Pressure Pain Threshold and Thermography: A Single Blinded Randomized Clinical Trial

**DOI:** 10.3390/jcm8101632

**Published:** 2019-10-05

**Authors:** María Benito-de-Pedro, Ricardo Becerro-de-Bengoa-Vallejo, Marta Elena Losa-Iglesias, David Rodríguez-Sanz, Daniel López-López, Julia Cosín-Matamoros, Eva María Martínez-Jiménez, César Calvo-Lobo

**Affiliations:** 1 Facultad de Enfermería, Fisioterapia y Podología. Universidad Complutense de Madrid, 28040 Madrid, Spainjuliacosin@hotmail.com (J.C.-M.); eva.hache2@hotmail.com (E.M.M.-J.);; 2 Faculty of Health Sciences, Universidad Rey Juan Carlos, 28922 Alcorcón, Spain; marta.losa@urjc.es; 3 Research, Health and Podiatry Unit. Department of Health Sciences. Faculty of Nursing and Podiatry, Universidade da Coruña, 15403 Ferrol, Spain; daniellopez@udc.es

**Keywords:** acupressure, myofascial pain syndrome, musculoskeletal diseases, trigger points

## Abstract

Background: Deep dry needling (DDN) and ischemic compression technic (ICT) may be considered as interventions used for the treatment of Myofascial Pain Syndrome (MPS) in latent myofascial trigger points (MTrPs). The immediate effectiveness of both DDN and ICT on pressure pain threshold (PPT) and skin temperature of the latent MTrPs of the triceps surae has not yet been determined, especially in athletes due to their treatment requirements during training and competition. Objective: To compare the immediate efficacy between DDN and ICT in the latent MTrPs of triathletes considering PPT and thermography measurements. Method: A total sample of 34 triathletes was divided into two groups: DDN and ICT. The triathletes only received a treatment session of DDN (*n* = 17) or ICT (*n* = 17). PPT and skin temperature of the selected latent MTrPs were assessed before and after treatment. Results: Statistically significant differences between both groups were shown after treatment, showing a PPT reduction (*p* < 0.05) in the DDN group, while PPT values were maintained in the ICT group. There were not statistically significant differences (*p* > 0.05) for thermographic values before and treatment for both interventions. Conclusions: Findings of this study suggested that ICT could be more advisable than DDN regarding latent MTrPs local mechanosensitivity immediately after treatment due to the requirements of training and competition in athletes’ population. Nevertheless, further studies comparing both interventions in the long term should be carried out in this specific population due to the possible influence of delayed onset muscle soreness and muscle damage on PPT and thermography values secondary to the high level of training and competition.

## 1. Introduction

Myofascial Pain Syndrome (MPS) comprises a set of motor, sensory and autonomic symptoms and signs caused by the presence of myofascial trigger points (MTrPs) [1]. MTrPs may be considered as sensitive points located in muscular strained bands [2], which may be commonly presented in general consultations with a prevalence of around 30% of patients, being even more frequent in tertiary pain clinics [3]. The identification of these MTrPs by physical examination showed a good inter-examiner reliability [4]. These points may be divided according to their clinical activity into active and latent MTrPs. Most common symptoms associated with the presence of MPS were local and/or referred pain, motor dysfunction of the affected muscle, restricted range of mobility, fatigue, weakness, or reduced coordination [5]. Latent MTrPs seem to present all characteristics of active MTrPs, although usually with a lower degree of sensitization [6,7]. Latent MTrPs do not present spontaneous pain, while active ones always present spontaneous pain [8,9]. In addition, local sensitivity and referred pain of latent MTrPs seems to only be maintained during mechanical stimulation [10,11,12,13]. 

Triathlon may be considered a new sport modality on the rise, reaching up to around 3.5 million participants around the world [14] and including orderly swimming, cycling and running [14]. Sport may produce many health and even social benefits, but it usually involves a risk of injury [15,16]. Injuries seem to be characterized by cutting off triathletes’ training or competitions during a variable interval of time [17].

In 2014, Bertola et al. published a study on 190 triathletes showing that the most frequent lesion kind was the muscular type and the highest number of lesions during the study was located in the triceps surae region, reaching up to 39% prevalence [18]. In 2013, the study carried out by Andersen et al. agreed that the triceps surae was the body area which was the most injured region in a period of 24 weeks before and after competition [19].

The sports activity level increase of an athlete may imply a parallel growth of the associated risk factors, both extrinsic and intrinsic factors, for the appearance of lesions [20], and among others, the so-called MPS.

MPS is mainly produced by traumatisms or micro-trauma of repetition and muscular overuse [21,22], which may be coincident with a very high percentage of injuries in triathlon [23]. MPS may be developed during sports activities if the use of the muscle exceeds its capacity and its normal recovery is disturbed [1]. In addition, this condition may appear if a weak muscle is overloaded in an attempt to perform normal activity without presenting the necessary muscle preparation [5].

Active and latent MTrPs region present a lower pressure pain threshold (PPT) than the normal sites, as well as active MTrPs show a lower PPT than latent MTrPs [8]. Latent MTrPs’ presence in the triceps surae may present a clinical significance related to gait cycle propulsion and future dysfunctions of the triceps surae [24].

Two treatments may be proposed for this syndrome. Among the non-pharmacological options, deep dry needling (DDN), despite the controversy, is currently considered one of the most effective interventions for the direct inactivation of MTrPs [25] and is gaining attention for the treatment of MTrPs [26,27]. In addition, ischemic compression (ICT) may be considered another effective intervention for MPS treatment. Prior reviews have shown moderately strong evidence for the use of ICT in order to produce immediate symptomatic relief of MTrPs [28,29]. In relation to the thermographic measurement of the MTrP area where the treatment is carried out, several studies have shown that both DDN [30] and ICT [31] may improve blood circulation after interventions generating an associated thermal change of the intervention region. Skin temperature control may be a function of blood flow, which may be controlled by the autonomic nervous system [32]. Central control of skin temperature may affect both sides of the body uniformly and simultaneously [32,33,34]. Despite the fact that the literature clearly documents that there is not good evidence for thermography as a valuable tool for detecting long term MTrPs in the region of interest [35], this tool may be an excellent instrument for the monitoring of neuromusculoskeletal alterations and evaluation of MTrPs interventions in the short term [36], including invasive procedures [37]. In relation to pressure pain threshold (PPT), both DDN [38,39] and ICT [28,29] seem to produce an improvement of the symptomatology in the short term. 

Both DDN and ICT have previously been shown to increase PPT versus controlled placebo or sham interventions immediately after these treatments [40,41,42]. Nevertheless, a prior study showed a greater PPT increase in MTrPs of the trapezius muscle for patients treated with DDN with respect to ICT at 48 h after treatment, but statistically significant differences were not found between both groups for PPT measurements immediately after treatment [43]. In addition, both DDN and ICT may influence skin temperature of the treated MTrP area, but there are no prior studies comparing both interventions in order to evaluate their thermographic effects [30,31]. Indeed, triathletes need to be commonly treated during competition or training [14,17], showing a high frequency of MTrPs presence in the triceps surae [18,19,20], and both DDN and ICT may be effective techniques versus controlled placebo or sham interventions immediately after treatment [40,41,42]. To date, there is a lack of research regarding the efficacy of DDN as an invasive procedure, compared to ICT, as a conservative treatment, in latent MTrPs located in triceps surae of triathletes immediately after treatment [40,43], to verify if the results comparing both interventions in the trapezius muscle [44] can be extrapolated due to the features of the triathletes population in the short term.

Therefore, the aim of this randomized clinical trial was to determine the immediate effects of the DDN versus ICT, mainly related to the PPT and skin temperature in the latent MTrPs of the triceps surae from triathletes. We hypothesized that triathletes receiving DDN would exhibit greater improvements in PPT and greater temperature increase than those triathletes receiving ischemic compression.

## 2. Methods

### 2.1. Design

A secondary analysis of a single blinded randomized clinical trial was carried out. The evaluator was blinded in order to assess the immediate effectiveness of a single treatment session of DDN versus a single intervention session of ICT in the latent MTrPs of triceps surae of triathletes. The primary outcome for this secondary analysis was PPT over the treated MTrPs. The secondary outcome measurement was the skin temperature of the treated MTrPs. Both measurements were carried out before and after 5, 10, 15, 20, and 25 min of the treatment session according to our prior published randomized clinical trial protocol [45]. In addition, a reliability analysis was carried out for each outcome measurement according to our protocol. Indeed, all measurements were carried out 24 h after the last training/sport session of the evening. This randomized trial was prospectively registered at Clinicaltrials.gov with the clinical trial number: NCT03273985. The study was approved by the human research committee of the Hospital Clinico San Carlos, Madrid-Spain (CEIC Hospital Clínico San Carlos 02/17), and all subjects signed the informed consent form before participation in the study.

### 2.2. Participants

Participants in this study were triathletes who carried out 15–20 h of weekly training and presented a minimum experience of 3 years practicing medium-distance triathlon (1.9 kilometers of swimming, 90 kilometers of cycling, and 21 kilometers of running) [14]. Triathletes were recruited from the podiatric and physiotherapy clinic of Fisiofuenla s.l.p from September to December 2017, according to a randomized sampling method and after identifying the eligible population by a clinical exploration carried out by the principal investigator in order to determine the presence of latent MTrPs in the triceps surae and identify among them the selection criteria. Necessary inclusion criteria for the participation of triathletes in the study were: 1. Presence of a knot in the palpable taut band of skeletal muscle; 2. Presence of a hypersensitive point in the taut band; 3. Patients who reported local or referred pain in the area of the latent MTrP after mechanical stimulation. The following criteria excluded the triathletes from the study: 1. Age outside the range of 18–75 years; 2. Lower limbs neurology disorders denoted by the DN4 questionnaire [46]; 3. Positive cognitive screening according to the Pfeiffer questionnaire [47]; 4. In-take or injected anti-coagulant or anti-aggregant medication; 5. Presence of prosthesis in the lower limb; 6. Systemic or local infection in the lower limb; 7. Fibromyalgia, autoimmune disease, iron deficit, or hypothyroidism; 8. Presence of fear of needles.

### 2.3. Simple Size Calculation 

A sample size calculation was carried out by the difference between two groups using the software of G*Power 3.1.9.2 (G*Power^©^, Dusseldorf university, Dusseldorf, Germany) considering the latent MTrP PPT of the triceps surae muscle measured immediately after treatments of a pilot study (*n* = 10) with two groups (mean ± SD), five triathletes in the DDN group (1.80 ± 0.37 kg/cm^2^) and five triathletes in the ICT group (2.46 ± 0.79 kg/cm^2^) [48]. Furthermore, a two-tailed hypothesis, an effect size of 1.06, a power (1-β error probability) of 0.80, α error probability of 0.05, and an allocation ratio (N2/N1) of 1 were applied for sample size calculation procedure. Thus, a total sample size of 30 triathletes, divided into 15 triathletes for the DDN group and 15 triathletes for the ICT group, was calculated, showing an actual power of 0.807. Finally, considering the possible 10% loss to follow-up, 34 triathletes, divided into DDN (*n* = 17) and ICT (*n* = 17) groups, were included as the used sample size.

### 2.4. Latent MTrPs Identification

Latent MTrPs were identified by the same clinician who carried out the outcome measurements before and after interventions and was blinded to treatment allocation. The presence of latent MTrPs was confirmed by the investigator who carried out both interventions. The most hyperalgesic latent MTrP of the triceps surae was selected and marked with a circle by a permanent marker in order to be evaluated and treated. This latent MTrP was defined as the most hyperalgesic nodule in a taut band generating local or referred pain under the same pressure of mechanical stimulation by manual palpation [49,50].

### 2.5. Primary Outcome: Pressure Pain Threshold

PPT may be defined as the pressure applied at the time when the patient’s sensation changes from pressure to pain [51]. PPT was evaluated in the selected latent MTrP using the Wagner analog algometer. This tool was a mechanical algometer with a rubber surface of 1 cm diameter at the tip of a piston, FDK/FDN series Force Dial, Wagner Instruments, 1217 Greenwich, and CT 06836, which presented excellent reproducibility, validity and reliability [52]. This tool was perpendicularly applied to the MTrP of each triathlete, increasing the pressure with a progression of 1 kg per second. The pressure stopped when the patient reported that the sensation changed from pressure to pain. The value of the pressure was recorded in kg/cm^2^.

The subjects were previously trained to indicate when the sensation changed from pressure to pain. A total of 10 measurements were performed for each muscle, five pre-intervention and five post-treatment, separated by 5 min. A high intra-examiner and inter-examiner reliability have been reported for the measurements carried out with the selected algometer (intraclass correlation coefficient from 0.80 to 0.97) in order to evaluate PTT over muscle tissue [53,54].

### 2.6. Second Outcome: Thermographic Measurement.

This measurement was performed with a thermographic camera with MSX technology, thermal image, and a resolution of 320 × 240 pixels. An automatic registry detected temperatures between 0.06 and 250 º by a camera with an infrared resolution of 240 × 320 pixels, inside a visual image, which facilitated the location of areas of interest during these measurements. Triathletes were warned to avoid exposure to the sun, body lotions, ingestion of vasodilators, and exercises and severe activities on the day of the thermographic evaluation [55]. Triathletes waited in prone position, with bare legs at 24.1 ± 1 °C and a humidity of 45% ± 10% in the center room where the study was carried out [55]. The thermal activity was studied in an area of 1 cm^2^ located in the central part of the MTrP and its same symmetrical point in the opposite part of the body; this variable was registered in degrees Celsius. Triathletes were asked to be placed in prone position on a treatment table. In order to acclimate to the conditions of the laboratory, triathletes were placed in this way in the treatment room 10 minutes before starting these measurements [56]. The environmental factors such as light and temperature were kept constant for normalization. These measurements were performed five times before and five times after treatment. After 10 minutes in the described position, the first temperature measurement was carried out determining two points, one point coinciding with the latent MTrP location and another point coinciding with the same anatomic location with healthy soft tissue in the contralateral limb. All evaluations were carried out by the same examiner [56].

### 2.7. Treatment Allocation 

Since both DDN and ICT have previously been shown to increase PPT versus controlled placebo or sham interventions immediately after these treatments [40,41,42], this study was not controlled and both interventions were compared in an isolated manner without including a control group. Prior to distributing all study subjects between both groups, an external researcher collected the necessary data for each triathlete. After, a randomization was carried out dividing the sample into DDN or ICT groups. This procedure was performed with the statistical and epidemiological analysis system Epidat 4.2. Individual and numbered sheets were prepared sequentially with a randomized assignment and placed in sealed opaque envelopes. A second investigator was in charge of opening the envelopes. Each outcome measurement was measured five times before and after the intervention by an independent investigator.

### 2.8. Invasive Procedure: DDN Group

The DDN group of triathletes in this study received only one session of DDN, with disposable stainless steel needles (0.3x50 mm, Agupunt, Madrid, Spain). After the MTrP was located within the taut band, these needles were introduced in them [57]. In 1997, Hong described the “fast in, fast out” technique, which was selected to carry out the DDN treatment [58]. First, the area was cleaned by the therapist with alcohol. After, sterile gloves were used. Then, the needle was firmly held between the thumb and the second finger and was deeply inserted, penetrating through the MTrP [59]. The needle was moved up and down, without going out of the skin. Finally, the DDN technique was applied up to the limit of tolerance of the triathlete or until it reached the maximum number of 8–10 insertions [40].

### 2.9. Conservative Treatment: ICT Group

Pressure was applied through the thumb in the MTrP until the athlete’s pain threshold coinciding with his/her sensation change from pressure to pain. This pressure was maintained for 90 seconds [2].

### 2.10. Statistical Analysis

Statistical analyses were performed using the statistical software IBM SPSS (version 19.0, IBM Corp., Armonk, NY, USA). The 95% confidence intervals for each variable and the mean ± SD were calculated. The Shapiro-Wilk test was performed due to the size of each group being lower than 30 subjects, determining if there was a normal distribution in the quantitative variables of our study. Student’s t parametric test was used for the variables that were adjusted to the normal distribution (*p* > 0.05). Non-parametric Mann-Whitney *U* test was used for the variables that were not adjusted to the normal distribution (*p* < 0.05). Both tests were used to evaluate the differences between both groups of treatment as independent samples. All analyses were considered statistically significant with *p* <0.05 for a confidence interval (CI) of 95%.

In addition, reliability analyses were carried out for outcome measurements according to the explained protocol [45]. Intraclass correlation coefficients (ICC), lower and upper limits of the 95% CI, standard errors of measurement (SEM), and Minimum Detectable Changes (MDC) were calculated. ICC values were categorized as poor with an ICC lower than 0.40, fair with an ICC from 0.40 to 0.59, good with an ICC from 0.60 to 0.74, and excellent with an ICC from 0.75 to 1.0 [60]. SEM values were calculated in order to measure the error range of each parameter by the formula SEM = SD × √(1-ICC). MDC values were calculated in order to determine the change magnitude necessary to provide confidence changes without the influence of measurement errors or random variations by the following formula MDC = √2×1.96×SEM. Both SEM and MDC were analyzed according to Bland and Altman [61].

## 3. Results

**Figure 1:**
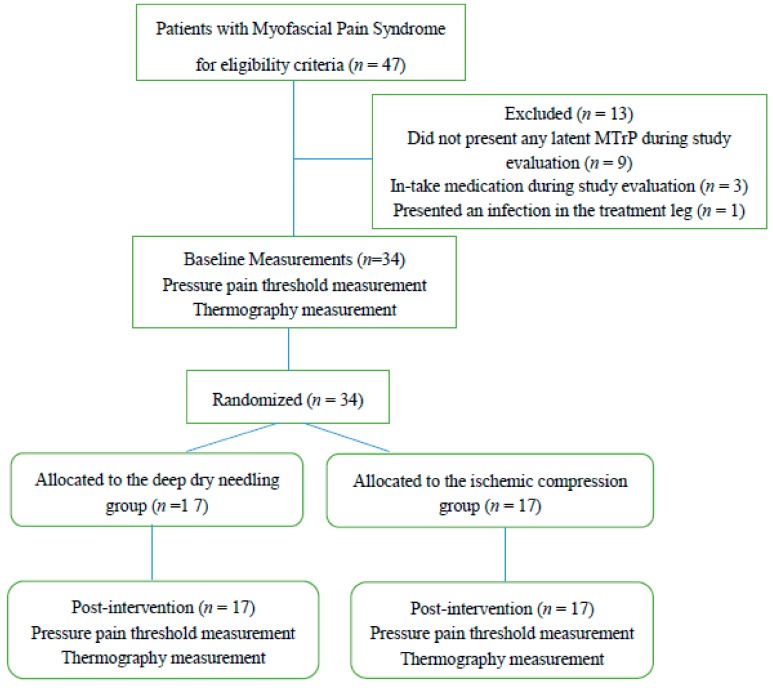
Flow diagram of patients throughout the course of the study.

Out of a total of 47 individuals recruited, 13 subjects were excluded from the study, n = 9 subjects because they did not present MTrPs during evaluation, *n* = 3 subjects because they took the medication during the study course, and *n* = 1 subject due to a leg’s infection located in the leg during the study evaluation. Subjects did not present any adverse effects (Figure 1).

### 3.1. Sociodemographic Characteristics Attending to the Division by Treatment Groups

Table 1 showed the demographic data of the studied sample divided into both groups. Data of the DDN group, ICT group, and total sample were expressed as the mean ± SD and 95% CI of each one of the variables. The variables of age, height, weight, size of footwear, and BMI were compared according to the type of treatment received by the participants, finding that there were no statistically significant differences. 

### 3.2. Pressure Pain Threshold

Table 2 shows that there are no statistically significant differences before treatment between DDN and ICT groups, but after treatment there are statistically significant differences (*p* > 0.05), showing a lower PPT immediately after receiving treatment in athletes who received DDN with respect to ICT.

### 3.3. Thermography

Table 3 shows that there are no statistically significant differences (*p* > 0.05) for thermography measurements between both treatment groups, DDN and ICT, neither in the superficial zone to the latent MTrP nor in the same anatomic location with healthy soft tissue in the contralateral limb before and after treatment. 

### 3.4. Reliability Analysis

PPT measurements showed excellent reliability with ICC of 0.979 (lower and upper limits of the 95% CI from 0.965 to 0.988), SEM of 0.094 kg/cm^2^, and MDC of 0.260 kg/cm^2^. Also, thermography measurements showed an excellent reliability with ICC of 0.970 (lower and upper limits of the 95% CI from 0.951 to 0.983), SEM of 0.308 °C, and MDC of 0.854 °C. 

## 4. Discussion

DDN effectiveness remains controversial, while in some studies beneficial effects have not been observed [62,63], in others its effectiveness has been demonstrated [39]. In the reviewed studies including MTrPs DDN treatments, marked symptoms improvement was described [62,64]. Despite the controversy, DDN may be currently considered as one of the most effective techniques for direct MTrPs inactivation [25] and thus DDN use is increasing for the treatment of MTrPs [27,65].

Even though the DDN action seems to still not be known exactly [26,27], the most used theory explained that the mechanical effect of DDN may increase the blood flow in the muscle and therefore its oxygenation [38,66]. 

With respect to the effectiveness of ICT, a recent review regarding ICT use has shown moderate to strong evidence for the immediate symptomatic relief of MTrPs [28,29]. Certain studies have shown an increase in blood flow in the area where the technique was performed [31], for approximately 20 min.

The measurement of the temperature was carried out in the superficial zone of the latent MTrPs and their symmetrical location of the contralateral limb including healthy soft tissue. Based on the results obtained, the temperature of both areas was similar before and after treatment. Indeed, immediately after receiving the treatment, subjects who underwent DDN presented a similar temperature in the area of latent MTrPs with respect to the temperature prior to receiving this intervention and the temperature of the latent MTrPs which received ICT. 

Currently, there is no agreement about skin temperature patterns according to the presence of MTrPs [35]. Several authors have affirmed that the skin temperature of the MTrP area should not be used to detect or diagnose MTrPs. Measurement with the thermovision technique of active MTrPs located in the gluteus minimus indicated an increase in their temperature after treatment with DDN in patients with chronic sciatica [67]. Previously, Skorupska et al. [68,69,70] found that the presence of short-term vasodilatation after DDN suggested the involvement of sympathetic nerve activity in the myofascial pain presented in patients with sciatica. In 2013, Moraska [31] described an increase in blood flow in the MTrP area of around 20 minutes after ICT intervention, while this increase in blood flow lasted up to 30 min post-treatment after DDN intervention [66]. 

The MDC applicable to infrared thermography varied from 0.11 to 0.78° degrees Celsius according to de Jesus Guirro et al. [71] and was set at 0.85° degrees Celsius according to the reliability analysis of our thermography measurement protocol. Thus, in this study, the difference in Celsius degrees did not reach the MDC, and again the differences were not statistically significant.

The effectiveness of DDN compared to ICT in the upper trapezius was carried out in 2016 in a comparative study. The results at 48 h were better for DDN compared to ICT in terms of pain intensity reduction and increase in PPT [72,73,74,75,76]. Indeed, PPT were considerably greater after 48 h in those subjects treated with DDN than in those subjects who received ICT [43]. Coinciding with our study’s protocol, most cited DDN studies used the “Hong’s fast-in and fast-out” technique applied up to the limit of tolerance or when the maximum number of 8–10 insertions was reached [40,45]. In addition, ICT was applied in the MTrP until the athlete’s pain threshold coincided with sensation change from pressure to pain during 90 s according to prior recommendations and studies using the same pressure and duration [2,43,77]. Nevertheless, prior ICT recommendations provided two alternative treatment modalities using both a low pressure (under pain threshold) and long duration of 90 s or a high pressure (between pain and tolerance thresholds) and short duration of 30 s for immediate pain relief as well as MTrP sensitivity improvement [78].

The results of our study did not show statistically significant differences between both groups prior to treatment, however, statistically significant differences were found for PPT after DDN treatment, being almost 0.4 kg/cm^2^ lower than the PPT of the group treated with ICT.

The MDC applicable to the algometer was set at 0.09 kg/cm^2^ according to Mutlu & Ozdincler [79] and 0.26 kg/cm^2^ according to the reliability analysis of our PPT measurement protocol. Regarding our study findings, the obtained statistical differences between the DDN group and ICT group post-treatment exceeded these MDC values. Therefore, these differences were interpreted as secondary to the treatment and not to the measurement errors of the algometer and PPT measurement protocol. 

After this study, ICT may be more advisable for triathletes’ triceps surae latent MTrPs treatment due to the immediate PPT reduction with the DDN technique compared to the ICT intervention and the inherent risks secondary to an invasive technique [80,81]. Regarding the thermographic results, the absence of differences between both treatment groups in terms of surface temperature in the treatment area of the latent MTrPs and the consequent DDN risks that may arise, as previously discussed with invasive techniques, could suggest that ICT is a better intervention in athletes, although further studies are necessary in this field.

## 5. Limitations

Only immediate results were assessed. The possible increase of the PPT after DDN intervention was not observed in the short, medium or long term. The same observations should be applied to the thermographic response in the latent MTrPs. Thus, it is recommended to carry out measurements after the increase in blood flow, as previously commented.

Despite the fact that the duration, schedule and intensity of the training and sport sessions were similar for all triathletes, showing similar basal conditions for both groups, as well as the fact that measurements were performed in similar conditions (24 h after the last training at evening), the presence of delayed onset muscle soreness, which may also be associated with muscle damage peaking at 24–48 h from activity, could have influenced our measurements, producing a decreased PPT in muscles and skin temperature modifications [82]. Consequently, in spite of similar PPTs and skin temperature in the two groups before treatment, the muscle reactivity to the two applied techniques could have been different depending on the reasons for basal hyperalgesia. The PPT measurements of the latent MTrPs in our sample were lower than the PPT that showed over normal muscles [83]. In addition, future studies should evaluate all muscle surfaces, not only the latent MTrPs region, according to the muscle damage secondary to training and sport sessions in triathletes.

## 6. Conclusions

The findings of this study suggested that ICT could be more advisable than DDN regarding latent MTrPs local mechanosensitivity immediately after treatment due to the requirements of training and competition of the athletes’ population. Nevertheless, further studies comparing both interventions in the long term should be carried out in this specific population due to the possible influence of delayed onset muscle soreness and muscle damage on PPT and thermography values secondary to the high level of training and competition.

## Figures and Tables

**Table 1:** Sociodemographic characteristics attending to the division by treatment groups.

	DDN Group (*n* = 17)	ICT Group (*n* = 17)	*p*-Value
Age (years)	35.29 ± 5.39 (32.73–37.85)	33.76 ± 5.76 (31.02–36.50)	0.215
Weight (kg)	65.17 ± 10.71 (60.08–70.27)	69.17 ± 10.66 (64.10–74.24)	0.141
Height (cm)	170.35 ± 12.94 (164.19–176.50)	174.94 ± 6.96 (171.62–178.25)	0.103
BMI (kg/m^2^)	22.37±1.92 (21.46–23.29)	22.48 ± 2.35 (21.36–23.6)	0.443
Size of footwear (cm)	41.55±3.26 (40.00–43.11)	41.35 ± 2.73 (40.05–42.65)	0.421

Abbreviations: SD, Standard deviation; Kg, kilogram; cm, centimeter; m^2^, squared meter; BMI, body mass index; statistical significance at a *p*-value ˂ 0.05; 95% CI, Confidence Interval at 95%; DDN, deep dry needling; ICT, ischemic compression.

**Table 2:** Pressure pain threshold of the superficial area of the latent MTrP of the participants of both groups, before and after treatment.

	Before Treatment			After Treatment		
Variable	DDN group (*n* = 17)	ICT group (*n*= 17)	*p*-value *	DDN group (*n* = 17)	ICT group (*n* = 17)	*p*-value *
Pressure pain threshold	2.63±0.65 (2.32–2.94)	2.62 ± 0.66 (2.30–2.94)	0.485	1.94 ± 0.58 (1.66–2.22)	2.38 ± 0.73 (2.02–2.73)	0.032

Abbreviations: DDN, deep dry needling; ICT, ischemic compression; PPT, pressure pain threshold. * Parametric Student’s T test for independent samples; Statistical significance for a *p*-value ˂ 0.05.

**Table 3:** Thermography of the superficial area of the latent MTrP and the same anatomic location with healthy soft tissue in the contralateral limb before and after treatment.

	Before Treatment			After Treatment		
Variable	DDN group (*n* = 17)	ICT group (*n* = 17)	*p*-value **	DDN group (*n* = 17)	ICT group (*n* = 17)	*p*-value **
Temperature in superficial area of MTrP	31.73 ± 1.99 (30.78–3.67)	31.85 ± 1.71 (31.04–32.66)	0.422	31.71 ± 1.98 (30.77–32.65)	32.25 ± 1.47 (31.55–32.95)	0.186
Temperature in the contralateral limb	31.81±1.78 (30.96–32.66)	31.98±1.39 (31.31–32.65)	0.380	31.81 ± 1.75 (30.98–32.64)	32.22 ± 1.35 (31.57–32.86)	0.227

Abbreviations: DDN, deep dry needling; ICT, ischemic compression; MTrP, myofascial trigger point. ** Non-parametric Mann Whitney U test for independent samples; Statistical significance for a *p*-value ˂ 0.05.
